# Analysis of Knowledge, Attitude, and Practice of Self-Medication Among Students and Staff of a Caribbean Medical School

**DOI:** 10.7759/cureus.75646

**Published:** 2024-12-13

**Authors:** Ramesh Lolla, Chandramouli Krishna Kotakala

**Affiliations:** 1 Pharmacology, Trinity School of Medicine, St. Vincent, ABW; 2 Pharmacology, Maharaja's Institute of Medical Sciences, Vizianagaram, IND

**Keywords:** adverse effects, allopathic drugs, cross-sectional study, medical education, medical students, self-medication

## Abstract

Background

Self-medication is commonly practiced, especially among medical students, administrative staff, and faculty from preclinical and paraclinical departments, driven by accessibility, familiarity with medications, and perceived convenience. This study explored the incidence, patterns, and factors influencing self-medication within the Xavier University School of Medicine, Aruba, with a primary focus on medical students and administrative staff. The faculty included in the study were from preclinical and paraclinical departments such as anatomy, physiology, biochemistry, pathology, forensic medicine, microbiology, and community medicine.

Methods

A descriptive, cross-sectional study was conducted among medical students, administrative staff, and faculty from preclinical departments. Data were gathered through a pre-designed, standardized, and anonymous electronic questionnaire in English, which included both open-ended questions for qualitative insights and close-ended questions for structured quantitative analysis. Open-ended questions allowed participants to provide detailed, personalized responses, offering qualitative insights into their self-medication practices and related experiences. Closed-ended questions, on the other hand, provided structured answer choices, facilitating the collection of quantitative data for statistical analysis. This combination enabled a thorough exploration of demographic details, self-medication behaviors, and the factors influencing these practices.

Results

Allopathic medicines were the most frequently used, reported by 34 respondents (63%), primarily sourced from pharmacies by 43 respondents (79.6%). These medicines were commonly administered directly from bottles, spoons, or measuring devices, as reported by 37 respondents (68.5%). Saving time was the primary reason for self-medication, cited by 18 respondents (33.3%), followed by lack of insurance or prior familiarity with the medication, noted by nine respondents (16.7%). Adverse effects were reported by seven respondents (13%), with drowsiness and stomach pain being the most frequent issues. Risks such as delayed diagnosis and drug resistance were also mentioned. Despite these concerns, 40 respondents (74.1%) acknowledged that self-medication could be hazardous, yet 44 respondents (81.5%) continued the practice. Additionally, 13 respondents (24.1%) felt adequately educated to self-medicate, and 37 respondents (68.5%) supported incorporating self-medication education into formal medical curricula.

Conclusion

Self-medication was widespread among medical students, administrative staff, and preclinical and paraclinical faculty, driven by convenience and accessibility but accompanied by risks of adverse effects and delayed diagnosis. Integrating self-medication education into medical curricula and raising awareness can foster safer healthcare behaviors and improve outcomes.

## Introduction

Self-medication is a prevalent global concern, particularly prominent in developing and economically disadvantaged countries [1]. Patterns of self-administered pharmaceutical drug use are influenced by factors such as age, gender, education, socio-economic status, access to healthcare, and the perceived severity of illness [2,3]. While some view the high prevalence of self-medication positively, as a marker of self-reliance and awareness regarding personal health, it remains a double-edged sword [4].

Over-the-counter (OTC) drugs are often the most accessible, economical, and efficient intervention for individuals in communities with limited healthcare access [5,6]. Rational self-medication can alleviate the burden on healthcare systems, reduce waiting times, and lower costs in overburdened medical settings [7]. However, irrational self-medication poses significant risks, including delayed care-seeking behavior, missed diagnoses, delayed treatments, financial losses, increased disease morbidity and mortality, adverse drug interactions, treatment failures, and the development of drug resistance [8,9].

Although convenient, the practice of self-medication raises numerous concerns about medication safety. As Soon et al. highlight, the inappropriate use of drugs (e.g., adverse effects, interactions, and resistance) remains a significant issue [10]. Similarly, Loyola Filho et al. (2004) demonstrated that self-medication behaviors are often driven by sociocultural factors, access to medication, and individual perceptions of disease severity [11]. Ganguly et al. (2011) further emphasized that irrational self-medication, especially with antibiotics, poses a global threat by contributing to antimicrobial resistance. They stressed the importance of promoting the rational use of drugs through education and regulation [12]. These findings underscore the need for Caribbean medical schools to understand and address self-medication practices.

Numerous studies have examined the prevalence of self-medication in general populations and specific groups, such as healthcare students and professionals [13,14]. However, limited research exists on self-medication practices in the unique demographic and geographic setting of the Caribbean islands. In particular, there is no published data examining self-medication behaviors among professional and student communities in Caribbean-based medical institutions.

Establishing baseline data on self-medication in Aruba is critical for devising strategic interventions, updating essential medicine lists, and ensuring the safe use of OTC drugs [15,16]. Medical students, in particular, are inclined toward self-medication, driven by their perceived expertise in diseases and treatment modalities, along with convenient access to drug-related resources [16]. 

Thus, the present study aims to investigate the prevalence, patterns, and underlying factors influencing self-medication practices among medical students, faculty, and administrative staff in Aruba. By examining these behaviors in detail, the study seeks to identify common trends, motivations, and potential risks associated with self-medication within this unique demographic. The findings will provide critical insights that can guide the development of targeted interventions to promote safer self-medication practices. Additionally, the study aims to inform healthcare policymakers, enabling them to design strategies that address identified gaps, enhance education and awareness, and ultimately improve overall healthcare outcomes in the community.

## Materials and methods

Study design and setting

This descriptive, cross-sectional study was conducted at Xavier University School of Medicine, Oranjestad, Aruba. The study population comprised medical students, teaching faculty, and administrative staff. Data collection was carried out over a two-month period, from July to August 2023. The study was conducted with a sample size of 53 respondents, providing valuable preliminary insights into self-medication practices. At a 95% confidence level, assuming a prevalence of 50%, the sample size offers a margin of error of approximately ±13%. 

Data collection

A pre-designed, standardized, and anonymous questionnaire in English was used for data collection. The questionnaire was distributed electronically to informed and consenting participants. It included both open-ended and close-ended questions to gather comprehensive information on demographic, educational, and social variables, as well as self-reported therapeutic drug-seeking behavior and self-medication practices over the past five years. It included antibiotics, analgesics, non-steroidal anti-inflammatory drugs (NSAIDs), beta-blockers, antihypertensive drugs, diuretics, metformin, vitamins and supplements, and herbal remedies. This highlights the diversity of medications used and offers insights into the patterns and preferences in self-medication practices within the study population. The questionnaire was specifically designed to capture key aspects of self-medication, including its prevalence, common causes, and emerging trends. The questionnaire used in this study was designed based on a comprehensive review of existing literature and validated by a panel of experts to ensure its relevance and suitability for the study objectives. Internal consistency was assessed using Cronbach's alpha, which demonstrated a satisfactory reliability score (e.g., ≥0.7) [3,14].

Inclusion criteria

Participants were included in the study if they were medical students, faculty, or administrative staff affiliated with Xavier University School of Medicine, Aruba. Only those who provided written informed consent to participate in the study were eligible. The inclusion criteria also required participants to be available during the study period, conducted from July to August 2023, and to have completed the survey questionnaire. Additionally, participants reporting self-medication practices within the last five years were prioritized for inclusion to align with the study’s objectives.

Exclusion criteria

Individuals unwilling to provide written informed consent were excluded from participation. Additionally, those with medical conditions or comorbidities that could independently influence self-medication practices, such as chronic mental health conditions or cognitive impairments, were not considered for the study. Responses that were incomplete or inconsistent were excluded to ensure the quality and reliability of the data. Furthermore, individuals outside the target population, such as those not associated with the university as students, faculty, or administrative staff, were excluded from the analysis.

This thorough delineation of inclusion and exclusion criteria aimed to ensure that the study population was representative of the target demographic and that the results were both reliable and valid.

Data analysis

It was performed using SPSS software (version 26.0, IBM Corp., Armonk, NY). Descriptive statistics were applied to summarize demographic, educational, and occupational characteristics, with results presented as frequencies and percentages for categorical variables. The prevalence of self-medication practices and associated factors was analyzed quantitatively. Missing or incomplete data were excluded from the analysis to maintain the integrity of the results.

Ethical considerations

This study adhered to the ethical principles outlined in the Declaration of Helsinki. Ethical approval was obtained from the Institutional Review Board (IRB) of Xavier University School of Medicine under the approval number XUSOM/IRB/2023/06/001. Detailed information about the study was provided to all participants, and informed consent was obtained prior to their participation. The confidentiality and anonymity of participants’ information were strictly maintained throughout the research process.

## Results

A total of 54 participants provided responses, detailing demographic characteristics, self-medication practices, and associated behaviors. The self-medication practices reported included the use of antibiotics, analgesics, NSAIDs, beta-blockers, antihypertensive drugs, diuretics, metformin, vitamins, supplements, and herbal remedies. Key findings are summarized as follows: demographic characteristics - 24 respondents (44.4%) identified as male, 28 (51.9%) as female, and two (3.7%) preferred not to disclose their gender. A majority, 46 respondents (85.2%), were unmarried or divorced, while 8 (14.8%) were married. Regarding nationality, 22 respondents (40.7%) were from the United States, followed by 15 (27.8%) Canadians, and 17 (31.5%) from other countries (Table 1).

**Table 1:** Demographic characteristics of respondents It depicts the demographic distribution of participants (n=54) based on gender, marital status, and nationality. Percentages are provided for each category, including female (28 (51.9%)), male (24 (44.4%)), married (8 (14.8%)), unmarried/divorced (46 (85.2%)), and nationalities such as USA (22 (40.7%)) and Canada (15 (27.8%)).

Variable	Frequency (n=54)	Percentage (%)
Gender		
Female	28	51.9
Male	24	44.4
Prefer not to mention	2	3.7
Marital status		
Married	8	14.8%
Unmarried/divorced	46	85.2%
Nationality		
USA	22	40.7
Canada	15	27.8
Other countries	17	31.5

Educational and occupational profile

About half had a bachelor's degree, 25 (46.3%), while 11 (20.4%) had a postgraduate one. Most of the participants, 45 (83.3%), were medical students, while eight (14.8%) comprised faculty (Table 2).

**Table 2:** Educational and occupational profile *The medical faculty were from preclinical and paraclinical departments such as anatomy, physiology, biochemistry, pathology, forensic medicine, microbiology, and community medicine.

Variable	Frequency (n=54)	Percentage (%)
Educational qualification		
Bachelor’s degree	25	46.3
Postgraduate degree	11	20.4
Others	18	33.3
Occupation		
Medical students	45	83.3
*Faculty	8	14.8
Others	1	1.9

Regarding living arrangements, 9 respondents (16.7%) lived with other family members apart from the household head, 11 respondents (20.4%) lived in a household of three members, and 10 respondents (18.5%) in a household of four members. Household income ranged between USD 50,000 and 100,000 for 8 respondents (33.3%) and USD 100,000 or more for 14 respondents (25.9%). Additionally, 16 respondents (29.6%) chose not to disclose their income (Table 3).

**Table 3:** Living arrangements and annual household income

Variable	Frequency (n=54)	Percentage (%)
Family cohabitation		
Lives alone	9	16.7
With 3 family members	11	20.4
With 4 family members	10	18.5
Annual income (USD)		
Above 100,000	14	25.9
50,000-100,000	18	33.3
Prefer not to say	16	29.6

Self-medication practices

A minority, 9 (16.7%), reported taking no medication in the last year, and the majority, 45 (83.3%), reported self-medication within the past year. Saving time was the main reason people chose pharma apps, cited by 18 respondents (33.3%), followed by issues such as a lack of insurance or expensive treatments (9 respondents, 16.7%) and past prescriptions or experiences (7 respondents, 13%). The main reason for limited access to healthcare facilities was reported as distance, as noted by 21 respondents (38.9%) (Table 4).

**Table 4:** Self-medication practices *Antibiotics, analgesics, NSAIDs, beta-blockers, antihypertensive drugs, diuretics, metformin, vitamins, supplements, and herbal remedies

Variable	Frequency (n=54)	Percentage (%)
*Self-medication in the past year		
Yes	45	83.3
No	9	16.7
Reasons for self-medication		
Save time	18	33.3
No insurance or expensive treatment	9	16.7
Old prescription or prior experience	7	13.0
Doctor's clinic far or other reasons	21	38.9

Conditions treated with self-medication

The conditions reported were categorized based on their symptoms. Headache/migraine, 10 (18.5%), and cough/runny nose, 7 (13%), were among the most commonly reported issues. Sore throat, 8 (14.8%), and fever/body aches, 6 (11.1%), were also frequently mentioned, along with muscle and joint pain, 9 (16.7%), as other prevalent complaints (Table 5).

**Table 5:** Conditions treated with self-medication

Common illnesses	Frequency	Percentage (%)
Headache/migraine	10	18.5
Cough/runny nose	7	13.0
Sore throat	8	14.8
Fever, body aches	6	11.1
Muscle pain, joint pain	9	16.7

Medication choice and sources

Most often used were allopathic medicines, 34 (63%), followed by herbal/naturopathic remedies, 17 (31.5%). Pharmacies were the main source of medication in the urban area, 43 (79.6%), with the lower proportion indicating family, 9 (16.7%), or primary healthcare centers, 2 (3.7%) (Table 6). 

**Table 6:** Choice of medication

Medication type	Frequency (n=54)	Percentage (%)
Allopathic	34	63.0
Herbal/naturopathic	17	31.5
Homeopathic or others	3	5.6
Source of medication		
Pharmacy	43	79.6
Family/friends	9	16.7
Primary healthcare center	2	3.7

Perceptions and experiences of all respondents

The majority, 44 (81.5%), considered self-medication helpful, and 6 (11.1%) were unsure. But 7 (13%) of participants reported adverse effects, such as drowsiness, severe stomach pain, rash, nausea, and throat swelling. Table 7 shows that the remaining 47 (87%) did not report adverse effects.

**Table 7:** Perceptions and experiences of self-medication

Variable	Frequency (n=54)	Percentage (%)
Perceived benefits		
Helped	44	81.5
Not sure	6	11.1
Did not help	4	7.4
Adverse effects experienced		
Yes	7	13.0
No	47	87.0

Awareness of self-medication and education on self-medication

In fact, only 13 (24.1%) thought they got adequate teaching about self-medication in medical school, with an opposing 25 (46.3%). A total of 37 (68.5%) felt that self-medication should be taught in medical education, which could affect future clinical practices. Nine respondents (16.7%) did not think that the weather would change their behavior (Table 8). 

**Table 8:** Self-medication education and awareness

Variable	Frequency (n=54)	Percentage (%)
Taught adequately about self-medication		
Yes	13	24.1
No	25	46.3
Not sure	16	29.6
Belief in the need for formal education		
Yes	37	68.5
No	9	16.7
Not sure	8	14.8

## Discussion

This study reveals a high prevalence of self-medication among medical students, faculty, and administrative staff in Aruba. The findings align with previous studies by Alshogran et al. (2018) and Shankar et al. (2002), which reported self-medication as a widespread practice among university students and in community settings [14,15].

The observed high prevalence of self-medication in this study can be attributed to factors such as convenience, familiarity with drugs, and easy access to over-the-counter (OTC) medications. Common reasons for self-medication, such as saving time and difficulties in accessing healthcare services, are consistent with findings by Gupta and Chakraborty (2022), who identified similar motivations in urban communities [16,17]. Systemic barriers, including physician shortages and delays in obtaining appointments, may drive individuals toward self-reliance in managing health concerns, a phenomenon also noted by Rangari et al. (2020) in rural settings [18].

The study identified a range of conditions treated through self-medication, with headache/migraine, sore throat, and muscle/joint pain being the most commonly reported. These findings echo those in the literature, where similar conditions are frequently self-managed with allopathic medicines due to their availability and perceived effectiveness [14,16].

While 81.5% of participants perceived self-medication as beneficial, the associated risks cannot be ignored. About 13% of respondents experienced adverse effects, such as drowsiness and severe stomach pain, highlighting the need for rational medication use. Alshogran et al. (2018) emphasized that irrational self-medication poses risks such as delayed diagnosis, treatment failures, and drug resistance, all of which were acknowledged by 72.2% of participants in this study [14].

This study also highlights a significant gap in formal education on self-medication, with only 24.1% of respondents reporting that the topic was adequately covered during their training. Kafle et al. (2021) similarly observed inadequate education on rational drug use among medical students [17]. Integrating self-medication education into medical curricula could address misconceptions, promote evidence-based practices, and ensure that future healthcare providers responsibly manage their personal health while effectively counseling patients.

The findings have critical implications for healthcare policy and regulation. Addressing systemic issues such as healthcare accessibility and shortages of qualified professionals, as observed in Aruba, should be prioritized. Shankar et al. suggested that awareness campaigns and pharmacist-guided OTC dispensing could mitigate the risks of irrational drug use [15]. Tailored interventions, such as incorporating self-medication topics into medical education and raising public awareness about the dangers of unsupervised drug use, could play a pivotal role in promoting safe practices.

Strengths and limitations

This study provides baseline data on self-medication practices in a unique geographic and professional setting. However, self-reported data on adverse effects may introduce recall bias or underreporting. Additionally, the findings are not generalizable due to the small sample size. Future studies involving larger and more diverse populations could provide further insights into self-medication behaviors.

## Conclusions

This study reveals a high prevalence of self-medication among medical students, faculty, and administrative staff in Aruba, driven by factors such as time constraints, lack of insurance, high treatment costs, and dissatisfaction with prior medical consultations. While many participants viewed self-medication as convenient and beneficial, they also acknowledged its risks, including delayed diagnoses and the potential for drug resistance. The findings underscore the need to address a critical gap in formal education on self-medication within medical curricula. Incorporating structured training on this topic can empower future healthcare professionals to make informed decisions about their own medication use and effectively guide patients. By mitigating risks such as adverse effects and improper drug use, such initiatives can foster safer practices, improve healthcare outcomes, and promote evidence-based approaches to medication management for both practitioners and patients.
